# A Role for the Malignant Brain Tumour (MBT) Domain Protein LIN-61 in DNA Double-Strand Break Repair by Homologous Recombination

**DOI:** 10.1371/journal.pgen.1003339

**Published:** 2013-03-07

**Authors:** Nicholas M. Johnson, Bennie B. L. G. Lemmens, Marcel Tijsterman

**Affiliations:** Department of Toxicogenetics, Leiden University Medical Center, Leiden, The Netherlands; University of Washington, United States of America

## Abstract

Malignant brain tumour (MBT) domain proteins are transcriptional repressors that function within Polycomb complexes. Some MBT genes are tumour suppressors, but how they prevent tumourigenesis is unknown. The *Caenorhabditis elegans* MBT protein LIN-61 is a member of the synMuvB chromatin-remodelling proteins that control vulval development. Here we report a new role for LIN-61: it protects the genome by promoting homologous recombination (HR) for the repair of DNA double-strand breaks (DSBs). *lin-61* mutants manifest numerous problems associated with defective HR in germ and somatic cells but remain proficient in meiotic recombination. They are hypersensitive to ionizing radiation and interstrand crosslinks but not UV light. Using a novel reporter system that monitors repair of a defined DSB in *C. elegans* somatic cells, we show that LIN-61 contributes to HR. The involvement of this MBT protein in HR raises the possibility that MBT–deficient tumours may also have defective DSB repair.

## Author Summary

The genome is continually under threat from exogenous sources of DNA damage, as well as from sources that originate within the cell. DNA double-strand breaks (DSBs) are arguably the most problematic type of damage as they can cause dangerous chromosome rearrangements, which can lead to cancer, as well as mutation at the break site and/or cell death. A complex network of molecular pathways, collectively referred to as the DNA damage response (DDR), have evolved to protect the cell from these threats. We have discovered a new DDR factor, LIN-61, that promotes the repair of DSBs. This is a novel and unexpected role for LIN-61, which was previously known to act as a regulator of gene transcription during development.

## Introduction

DNA is maintained in the cell as chromatin: double-stranded DNA wrapped around core histone octomers to form nucleosome subunits. Chromatin folds into higher order structures depending on how tightly DNA is wrapped around the histones and how closely the nucleosomes interact [1]. Condensed chromatin acts as a physical barrier that restricts DNA access and therefore must be remodelled to enable various cellular processes such as gene transcription, DNA replication and DNA repair [2]. This is principally achieved by post-translational modification to the N-terminal tails of histones. One example of this is the methylation of lysine residues, which alters the degree of chromatin compaction and provides a binding site for the recruitment of non-histone proteins such as malignant brain tumour (MBT) domain proteins [2]. Once bound to histones, MBT domain proteins condense chromatin and repress transcription of target genes [3]. The MBT domain is a highly conserved motif of approximately 100 amino acids in length found throughout metazoans from *C. elegans* to humans [4].

Some MBT domain proteins act together with Polycomb group (PcG) repressor complexes that are best known for establishing and maintaining gene expression patterns during development [4]. The *C. elegans* MBT protein LIN-61 is also implicated in transcriptional regulation. It is a member of the synthetic multivulva (synMuv) class B group of proteins that act redundantly with synMuvA proteins to repress transcription of *lin-3* EGF and *lin-60* Ras [5]–[7]. Separate to its role within the synMuvB pathway, we found *lin-61* is also involved in maintaining genome stability. Worms depleted of *lin-61* have elevated rates of germline and somatic mutation, including small DNA insertions and deletions, but how LIN-61 maintains the genome fidelity was unknown [8]. Intriguingly, other MBT proteins have been shown to act as tumour suppressors: *lethal(3)malignant brain tumour* [*l(3)mbt*)] mutants of *Drosophila* develop malignant transformations of the adult optic neuroblast and ganglion mother cells of the larval brain [9]; furthermore, the human MBT domain genes *L3MBTL2*, *L3MBTL3* and *SCML2* are mutated in rare cases of medulloblastoma [10]. Also, depletion of L3MBTL1 (another LIN-61-related protein) causes genome instability [11]. Therefore it appears MBT proteins may have a general role in genome stability. It is not known how these proteins prevent tumourigenesis or protect the genome, but their ability to repress transcription likely plays a central role considering that the *l(3)mbt* malignancies of *Drosophila* ectopically express germline genes, the expression of which is required for tumour growth [12]. Preventing the expression of germline genes in somatic tissues may be a conserved function of MBT proteins because *lin-61* mutants also express germline genes in the soma in a temperature-dependent manner [13].

As well as regulating transcription, an increasing number of chromatin-remodelling proteins (including PcG proteins) have been found to act within the DNA damage response (DDR). These proteins accumulate at sites of DNA damage where they locally modify chromatin to allow the recruitment of DNA repair proteins [14]. In the present study we investigate the cause of genomic instability in *lin-61* mutants. We show that LIN-61 acts within the DDR where it is needed for efficient double-strand break (DSB) repair in both the germline and somatic cells of *C. elegans*. LIN-61 promotes DSB repair by homologous recombination (HR), but not the competing pathways, non-homologous end joining (NHEJ) or single-strand annealing (SSA). Despite the requirement for LIN-61 in HR, it is dispensable for meiotic recombination and the DNA damage checkpoints (cell cycle arrest and apoptosis) in the germline. We also use a novel GFP-based HR reporter assay that confirms LIN-61 is needed for HR. This reporter monitors the repair of a single defined DSB and is a new tool for measuring HR in *C. elegans* somatic cells. This is the first report demonstrating that an MBT protein promotes DNA repair and provides an explanation for why MBT-deficient cells have genomic instability.

## Results

### Genomic instability in *lin-61* mutants

**Figure 1:**
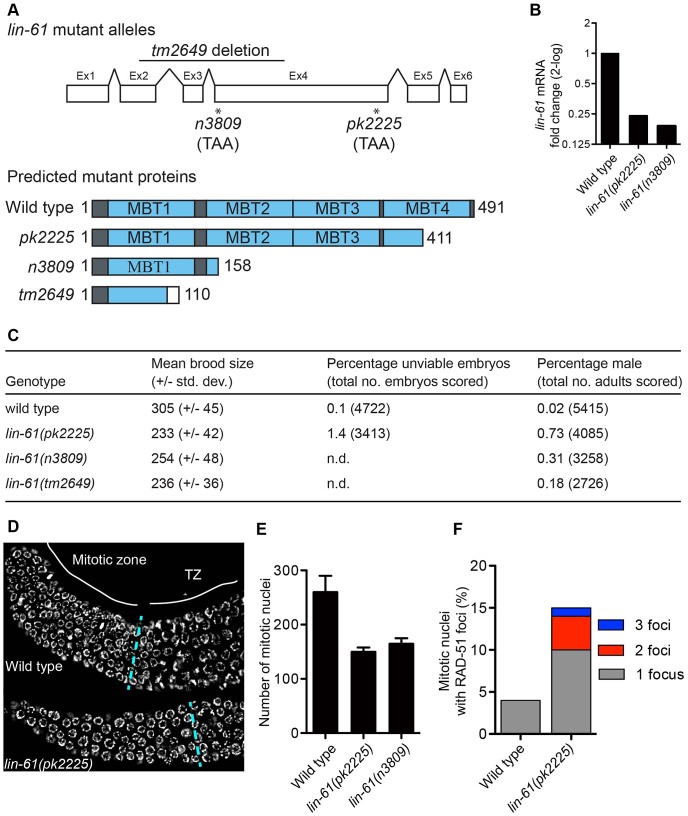
*lin-61* mutants display signs of genome instability and replication stress. (A) *lin-61* gene model (above) showing the location of *n3809*, *pk2225* and *tm2649* and predicted protein translations (below). Ex, exon. (B) Quantification of *lin-61* mRNA by qRT-PCR. Data is normalised to wild type. (C) Table listing brood sizes, including proportion of male progeny and unhatched embryos. n.d., not determined. (D) Dissected and DAPI-stained germlines from young adults. A single layer of nuclei is shown for clarity. The blue dashed line separates the mitotic zone from the transition zone (TZ). (E) Histogram depicting the average number of nuclei per mitotic zone. Error bars represent s.d. (F) Stacked histogram showing the percentage of mitotic nuclei containing RAD-51 foci.

To investigate how LIN-61 contributes to genomic stability, we obtained three independently generated null alleles of *lin-61* (*n3809*, *pk2225* and *tm2649*; Figure 1A and Text S1). The fourth MBT domain [essential for binding H3K9me2/3; [15]] is truncated or deleted in each of the mutant LIN-61 proteins. Moreover, *lin-61* mRNA is reduced approximately four-fold in *n3809* and *pk2225*, likely due to nonsense-mediated decay (Figure 1B). Each of the three mutants produced small broods (17–24% fewer progeny than wild types; Figure 1C). This can be symptomatic of genomic instability as DNA repair mutants such as *brc-1*, *rfs-1*, *blm-1* and *smc-5/-6* also have small broods [16]–[19]. In accordance with their reduced fecundity, *lin-61* mutants had considerably smaller germlines than wild types and contained fewer nuclei in the mitotic compartment (Figure 1D–1E). What is more, there were signs of DNA damage in these cells: their mitotic nuclei contained considerably more spontaneous RAD-51 foci than those of wild types (Figure 1F). RAD-51 is the DNA strand exchange protein, which accumulates at DSBs and blocked replication forks, and therefore is a marker for DNA damage [20]–[22].

### LIN-61 is required for resistance to ionizing radiation but dispensable for meiotic recombination

**Figure 2:**
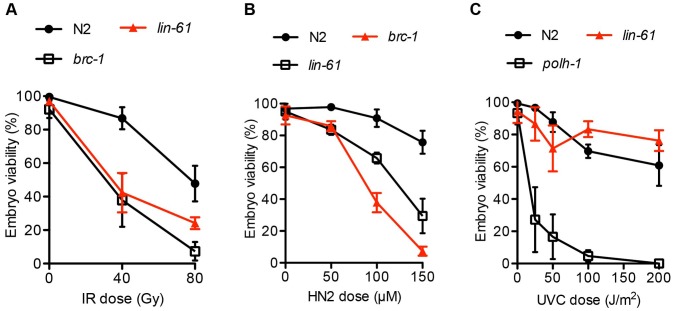
*lin-61* mutants are sensitive to IR and HN2, but not UV-C. L4 stage animals were challenged with (A) IR, ionizing radiation; and young adults were treated with (B) HN2, nitrogen mustard or (C) UVC, ultraviolet light subtype C. The average percentage of viable eggs is plotted. Error bars represent s.d.

Since *lin-61* mutant germ cells displayed genomic instability and signs of persistent spontaneous DSBs, we wondered whether *lin-61* mutants were sensitive to ectopically induced DSBs. We found that the germ cells of *lin-61* mutants were hypersensitive to ionizing radiation (IR), which is a potent inducer of DSBs (Figure 2A). Also primordial germ cells that are arrested in the G2 stage of the cell cycle in L1 stage larvae, are hypersensitive to IR in *lin-61* mutants animals (Figure S1).

The LIN-61 paralog, called MBTR-1 (Malignant Brain Tumour Repeat containing protein 1), shares a high degree of sequence conservation with LIN-61 and both proteins are comprised almost entirely of four MBT domains (Figure S2A). We wondered whether MBTR-1 too might be needed for resistance to IR-induced DSBs. To test this, we challenged *mbtr-1(n4775)* mutants with IR but found that they were not more sensitive than wild type controls (Figure S2B). Therefore LIN-61, but not the closely related MBT domain protein MBTR-1, is required for resistance to IR-induced DSBs in germ cells.

The IR-hypersensitivity of *lin-61* mutant germlines suggested that LIN-61 might be required for DSB repair during gametogenesis. We therefore investigated if LIN-61 also had a role in the repair of programmed DSBs that arise during meiosis. Meiotic DSB repair is required for the proper segregation of chromosomes to gametes and involves the repair of programmed DSBs introduced by the topoisomerase-like protein SPO-11 [23]. These DSBs are repaired by HR using the homologous chromosome as the repair template (interhomolog HR). The progression of DSB repair can be monitored in meiosis by following RAD-51 foci, which first appear at prophase, peak at early/mid-pachytene, and are resolved by late pachytene once DSB repair is completed [24]. The distribution of RAD-51 foci in *lin-61* meiotic cells was indistinguishable from those of wild types (Figure S3). This indicated that repair of SPO-11-introduced DSBs was unperturbed in *lin-61* mutants. Interhomolog HR enables crossover (CO) formation, which establishes the physical connection (chiasmata) that holds homologs together until their separation at the first meiotic cell division. Diakinesis stage oocytes of *lin-61* mutants contained the correct complement of six bivalents (paired homologs), which indicated that CO formation was competent in these mutants. Furthermore, *lin-61* mutants produced mostly viable progeny and did not display an increased incidence of males (Him) phenotype (Figure 1C). Failed meiotic recombination causes nondisjunction and aneuploidy due to the uncontrolled segregation of chromosomes to gametes, which manifests as embryonic lethality and the Him phenotype [25]. We conclude that LIN-61 is necessary for the repair of IR-induced DSBs but dispensable for CO formation and meiotic recombination. This phenotype is paralleled by the HR mutant *brc-1* and the cohesin-like mutants *smc-5/-6*. These mutants are IR hypersensitive due to defective DSB repair by HR that uses the sister chromatid (intersister HR) [19], [26], [27]. Our observation that *lin-61* mutants were hypersensitivity to IR suggested that LIN-61 might also contribute to intersister HR.

### *lin-61* mutants are hypersensitive to interstrand crosslinks but not UV lesions

In addition to repairing IR-induced DSBs, intersister HR is needed for repair of interstrand crosslinks (ICLs). ICLs are particularly cytotoxic lesions that block the replication fork by covalently linking opposing strands of double-stranded DNA [28]. During ICL repair, the crosslinked lesion is excised, thus producing a DSB substrate for intersister HR [29]. HR-deficient mutants like *brc-1*, or the *rad-51* paralog *rfs-1* are therefore hypersensitive to ICLs [21]. Consistent with LIN-61 having a possible role in intersister HR, we found that *lin-61* mutants were hypersensitive to nitrogen mustard (HN2), which is a potent inducer of ICLs (Figure 2B).

Other DNA lesions that block replication forks (such as bulky photoadducts made by UV light) do not cause a DSB and do not require HR for repair. Instead, translesion synthesis (TLS) DNA polymerases such as POLH-1 bypass these lesions to allow replication to proceed [30]. *polh-1* mutants are therefore hypersensitive to UV-C [31] but HR-deficient mutants such as *rfs-1* are not [21]. We found that *lin-61* mutants were not hypersensitive to UV-C (Figure 2C). The sensitivity of *lin-61* mutants to IR and HN2, but not UV-C, suggested that LIN-61 may promote DNA repair through HR, but is not required for the repair of other replication-blocking lesions such as photoadducts.

### LIN-61 has a role in HR, but not NHEJ, in somatic cells

**Figure 3:**
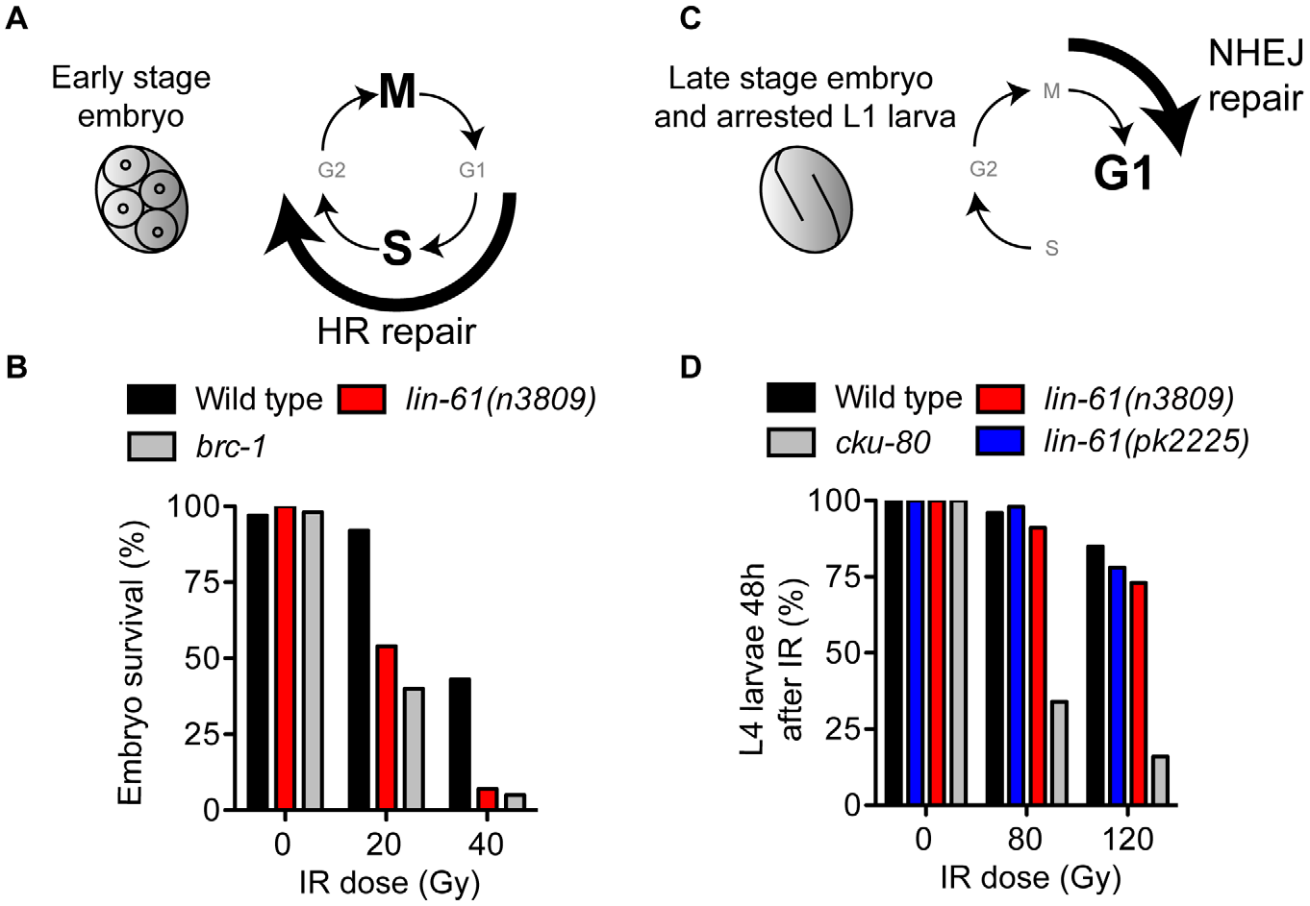
LIN-61 is needed for HR, but not NHEJ, in somatic cells. (A) Early stage embryos rapidly cycle between mitosis (M) and DNA synthesis (S), without gap phases (G1 and G2). HR is the prominent repair pathway in these cells. (B) Survival rates of IR-treated early stage embryos. (C) Most cells of late stage embryos and arrested L1 larvae are held in G1 phase. NHEJ is the principal DSB repair pathway in these cells. (D) The proportion of animals that developed to the L4 stage 48 hours after being γ-irradiated as L1 larvae.

LIN-61 is broadly expressed in somatic and germ cells throughout development [6]. To determine if LIN-61 contributes to DSB repair in somatic cells, as it does in germ cells, we used established assays that test the proficiency of HR, as well as the other major DSB repair route, NHEJ [32]. Somatic cells use either HR or NHEJ depending on developmental context and phase of the cell cycle. HR is active during S and G2 phases (when sister chromatids are closely aligned), whereas NHEJ can be performed throughout the duration of the cell cycle, but is especially important during G1 when HR is unavailable [33]. Early stage embryonic cells (<6 hours post fertilisation) rapidly transition between S phase and M phase, without G1 and G2 gap phases [34], [35] and are particularly reliant on HR for DSB repair [32] (Figure 3A). Accordingly, early stage embryos of HR-deficient mutants are very sensitive to IR, while those of NHEJ-deficient mutants are not [32]. To test whether *lin-61* promotes HR in somatic cells, we scored the viability of γ-irradiated early stage *lin-61* embryos. These embryos were indeed hypersensitive to IR, which was indicative of an HR defect (Figure 3B). Their degree of IR sensitivity was similar to that of HR-deficient *brc-1* embryos. While HR is the dominant DSB repair route in early embryos, NHEJ is the major repair pathway in late stage embryos and arrested L1 larvae because most of their cells are arrested in G1 [32] (Figure 3C). NHEJ-deficient L1 larvae have delayed or arrested growth in response to IR [32]. We found that wild type, *lin-61(n3809)* and *lin-61(pk2225)* L1 larvae did not display substantial growth delay following IR, whereas most NHEJ-deficient *cku-80* mutants failed to develop to the L4 stage 48 hours after irradiation (Figure 3D). L1 larvae of the HR-deficient mutant, *brc-1*, were also not hypersensitive to IR (Figure S4). Taken together, these results suggest that LIN-61 has a role in repairing DSBs by HR, but not NHEJ, in somatic cells.

### LIN-61 is not required for intersister HR in meiotic nuclei

**Figure 4:**
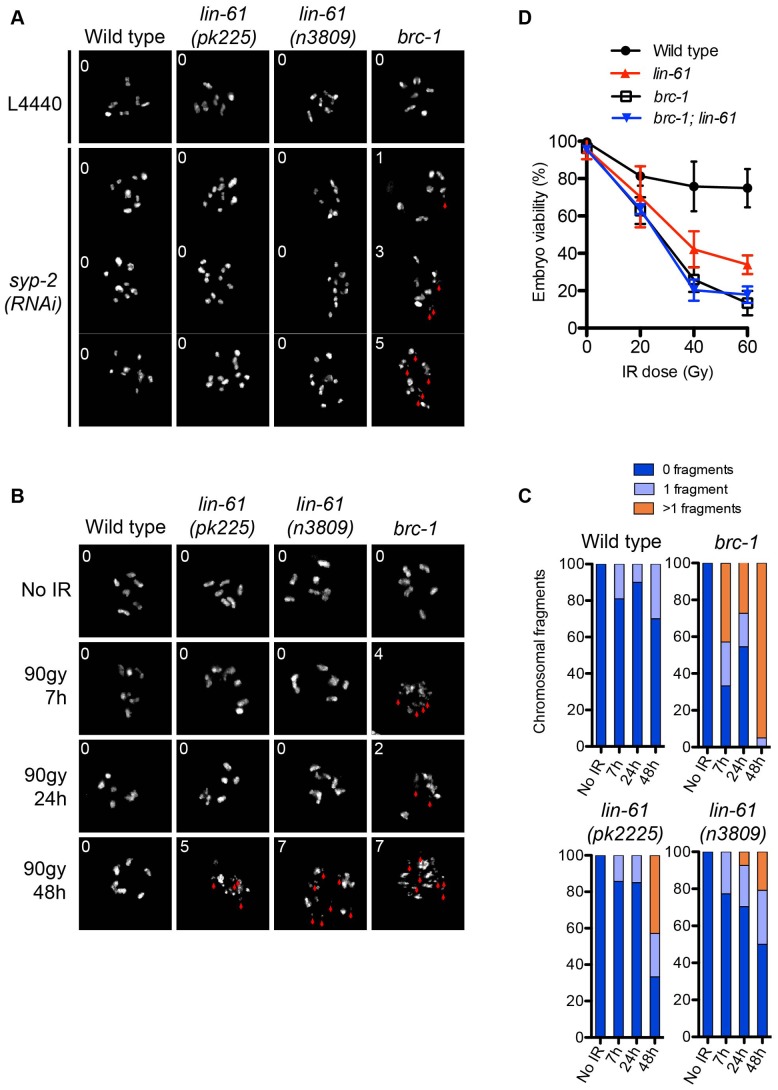
LIN-61 contributes to HR in mitotic cells, but is dispensable for meiotic HR. (A) DAPI-stained DNA bodies in diakinesis stage oocytes of animals mock treated (L4440) or depleted of SYP-2 by RNAi. (B) Time course of chromosomal fragmentation in response to 90 Gy dose of IR. In (A) and (B), the red arrowheads indicate chromosomal fragments and the inset number corresponds to the number of small fragments visible in the image. (C) Quantification of the chromosomal fragmentation. (D) Epistatic analysis of *brc-1* and *lin-61(pk2225)* IR sensitivity. L4 larvae were irradiated with the indicated dose. The percentage of viable embryos is plotted. Error bars represent s.d.

Although *lin-61* mutants phenocopy *brc-1* mutants in many aspects of genome stability, they also differ in some important aspects. For example, *brc-1* mutants display the Him phenotype, while *lin-61* mutants do not. Him is an indication of problems with chromosome segregation at meiosis. Like *brc-1* mutants, *lin-61* mutants are able to successfully complete meiosis, indicating that their interhomolog HR is proficient. However, by genetically disrupting the synaptonemal complex (SC), and thereby preventing interhomolog HR, it has been possible to demonstrate that BRC-1 contributes to meiotic intersister HR [27]. Adamo and colleagues observed that chromosomal fragments appear in the diakinesis stage nuclei of *brc-1* mutants that were depleted of key SC components [27]. Using this approach we tested whether LIN-61 also has a role in meiotic intersister HR. In contrast to *brc-1* mutants, neither the oocytes of *lin-61(pk2225)* or *lin-61(n3809)* contained chromosomal fragmentation after depletion of the core SC component, SYP-2 (Figure 4A). These data, together with those showing normal RAD-51 kinetics and successful chiasmata formation in *lin-61* mutants (Figure S3 and Figure 4A), indicate that LIN-61 is dispensable for HR in meiotic cells.

### LIN-61 contributes to DSB repair in mitotic germ cells but not meiotic germ cells

*lin-61* mutants are proficient in the repair, at meiosis, of SPO-11-introduced DSBs (using both intersister and interhomolog repair) but are hypersensitive to IR. To confirm that LIN-61 is required for DSB repair specifically in mitotic germ cells we used an assay that directly tests whether DSBs are adequately repaired in irradiated germ cells. Completion of DSB repair can be determined in germ cells by observing chromosomes at diakinesis because chromosome fragments are present if DSBs are unrepaired [36]. In the absence of exogenous damage, the diakinesis stage oocytes of *lin-61* mutants contained six bivalents and were not fragmented (Figure 4B). This demonstrated that DSBs induced by SPO-11 were efficiently repaired in *lin-61* mutants, as discussed earlier. Strikingly however, both *lin-61* mutants and the HR-deficient mutant *brc-*1 had severely fragmented chromosomes 48 hours after γ-irradiation (Figure 4B–4C). We anticipated that these nuclei could have been located within the mitotic zone at the time of irradiation, having subsequently migrated to the diakinesis stage 48 hours later. Failure to repair the introduced DSBs could therefore be due to defective HR whilst in the mitotic zone, or later whilst in the meiotic zone, or both. To distinguish between these possibilities we analysed earlier time points following irradiation (7 h and 24 h). For these time points, the nuclei being analysed were in meiosis when DSBs were introduced. We found that *brc-1* mutants had fragmented chromosomes at these earlier time points (7 h and 24 h) (Figure 4B–4C), which is consistent with BRC-1 acting in meiotic DSB repair [27]. In contrast, *lin-61* mutants, like wild types, rarely had fragmented chromosomes at early time points following irradiation (Figure 4B–4C). Thus while BRC-1 contributes to DSB repair in both mitotic and meiotic cells, LIN-61 seems to promote DSB repair only in mitotic cells. In accordance with that notion, we found that *brc-1* mutants were more sensitive to IR than *lin-61* mutants (Figure 4D). Moreover, *lin-61 brc-1* double mutants were no more sensitive to IR than *brc-1* single mutants suggesting that *lin*-61 acts within the *brc-1* genetic pathway (Figure 4D).

### LIN-61 is dispensable for RAD-51 focus formation

**Figure 5:**
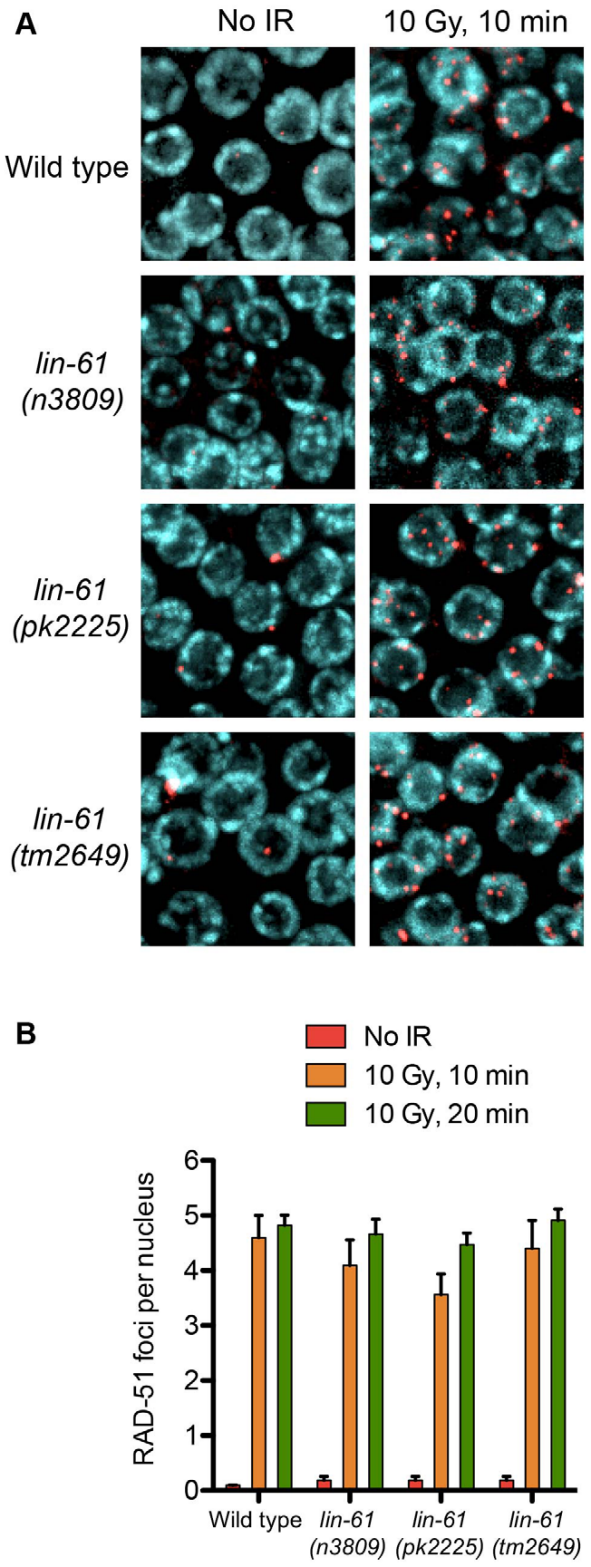
RAD-51 is loaded efficiently in irradiated *lin-*61 mutants. (A) RAD-51 foci (red) in mitotic nuclei (DNA is blue) of wild type and *lin-61* mutants 10 minutes after 10 Gy IR, or mock treatment. (B) Quantification of RAD-51 foci in mitotic nuclei. Error bars are S.E.M.

Having established that LIN-61 promotes DSB repair via HR, we looked to address which step of HR fails in *lin-61* mutants. The first stages of HR involve the nucleolytic processing at the DSB to expose single stranded 3′ overhangs (DNA end resection) and subsequent coating of these overhangs with RAD-51. RAD-51 foci rapidly formed in the γ-irradiated mitotic germ cells of both wild types and *lin-61* mutants (Figure 5A). Foci were detected at a very early time point after γ-irradiation (10 minutes), which showed that DNA end resection was unperturbed in these cells (Figure 5A). The loading of RAD-51 at SPO-11-induced DSBs was also normal in *lin-61* meiotic cells, as discussed earlier (Figure S3). Together this showed that DNA end resection at IR-induced and SPO-11-induced DSBs, as well as the loading of RAD-51 on resected DNA, was normal in *lin-61* mutants. The number of RAD-51 foci that formed in γ-irradiated germ cells was similar between wild types and *lin-61* mutants (4–5 foci per nucleus) (Figure 5B). Since the DNA in wild type and *lin-61* nuclei were equally susceptible to IR, the hypersensitivity of these mutants was not due to an elevated damage load.

### A novel GFP-based HR reporter system confirms that LIN-61 is required for HR in somatic cells

**Figure 6:**
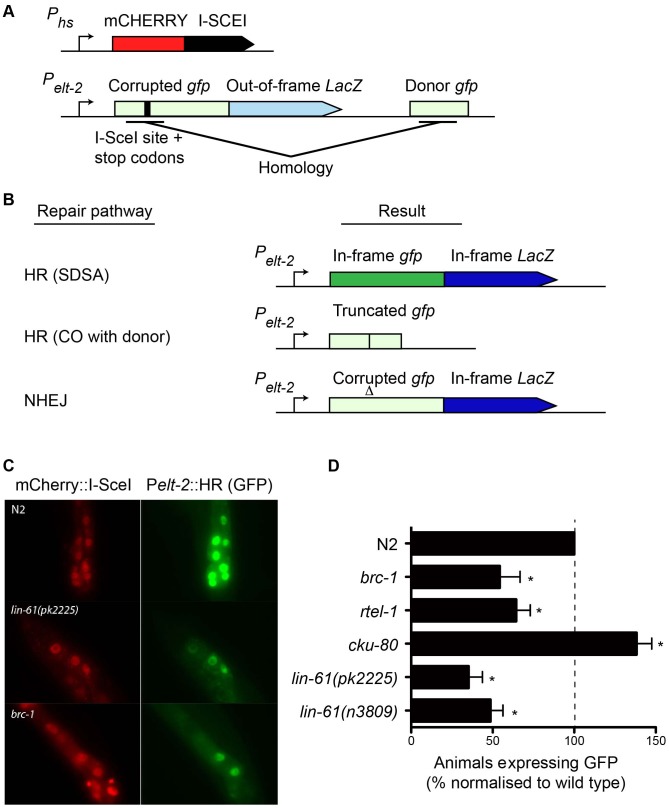
A novel GFP-based HR reporter system shows LIN-61 is needed for HR in somatic cells. (A) Schematic diagram of P*heatshock*::mCherry::I-SceI and the P*elt-2*::HR reporter. (B) Repair the of I-SceI-induced DSB can result in various outcomes depending upon which repair pathway is used. GFP expression is only restored by the HR-subpathway, synthesis-dependent strand annealing (SDSA). HR repair resulting in a CO between the reporter and the donor cannot restore GFP expression. Non-homologous end joining (NHEJ) cannot restore the *gfp* ORF, but can result in LacZ expression if stop codons are deleted. Light green and light blue represents out-of-frame/non-functional *gfp* and *LacZ*, respectively. Dark green and dark blue represents in-frame *gfp* and *LacZ*. (C) Images of mCherry::I-SceI (red) and GFP (green) expression in intestinal nuclei. (D) The percentage of animals with at least one intestinal nucleus expressing GFP after DSB repair. All data is normalised to N2 wild types (set to 100%). Average data from these experiments. Error bars represent s.d. * *p*<0.001.

While IR is a potent source of DSBs, it also causes oxidative damage to proteins and cell membranes [37]. To confirm that the hypersensitivity displayed by *lin-61* mutants was due to defective DSB repair (and not other types of damage), we developed an assay that specifically measures HR-mediated repair of a defined DSB. This assay was based on the DR-GFP reporter system, which has been used extensively to measure HR proficiency in cultured human cells [38]. Such an assay was previously unavailable to the *C. elegans* researcher. The new *C. elegans* reporter consisted of a *gfp* gene in which part of the open reading frame had been deleted and replaced by an I-SceI endonuclease recognition site, which rendered the GFP non-functional, and provided the defined location where the DSB could be introduced (Figure 6A). A fragment of *gfp* containing the sequences disrupted by the I-SceI site (but by itself non-functional) was located downstream of the reporter and served as a template for synthesis-dependent strand annealing (SDSA) (Figure 6A). SDSA is a sub-pathway of HR that results in gene conversion rather than a CO and is the most common HR pathway used to repair two-sided DSBs [39]. The reporter was designed such that repair of the DSB by SDSA (but not a CO pathway) would be able to restore expression to the corrupted *gfp* gene. Non-HR pathways such as NHEJ or SSA are unable to produce functional GFP (Figure 6B).

We created a transgenic strain that carried both the HR reporter and heat-shock inducible I-SceI endonuclease. I-SceI was fused to mCherry so that its expression could be easily monitored by epifluorescence. Since it is thought HR does not occur in postmitotic cells (i.e. G1/G0 stage cells), we chose to express the reporter in intestinal cells using the *elt-2* promoter as their nuclei undergo endoreplication (S phase without mitosis) at several points during post-embryonic development [40]. We first confirmed that induction of mCherry::I-SceI resulted in GFP expression. 60–80% of wild type worms expressed GFP in intestinal nuclei 24 hours after mCherry::I-SceI expression. Importantly, reporter activation was dependent upon DSB induction because non-heat shocked worms did not express GFP (data not shown). Also, GFP expression was dependent upon the donor *gfp* sequences since a disabled version of the HR reporter, which lacked these sequences, was not able to express GFP (Figure S5). To confirm that GFP expression depended on HR, we tested the effect *brc-1* mutation had on the reporter. BRC-1 promotes intersister HR in meiotic cells [27], and likely in somatic cells as well [41]. Indeed, *brc-1* mutants had significantly reduced frequency of HR reporter activation (Figure 6C–6D). This confirmed that the assay provided a measure of HR proficiency. We also used an *rtel-1* mutation to test whether reporter activation was dependent on the SDSA pathway. RTEL-1 is thought to influence HR pathway choice by removing the invaded DNA strand from its homologous template, which has the effect of promoting SDSA at the expense of CO outcomes [42]. The role of *rtel-1* in somatic cells was previously untested but we found that *rtel-1* mutants also had significantly reduced rates of HR reporter activation (Figure 6D). Therefore RTEL-1 likely promotes SDSA in somatic cells as it does in meiotic cells. A previous study showed that DSB repair pathways are dynamic and are in competition in *C. elegans* somatic cells such that the inhibition of one pathway caused increased activity in the others [41]. We therefore reasoned that inhibiting NHEJ should increase the frequency of HR reporter activation. As predicted, blocking NHEJ by *cku-80* mutation resulted in substantial elevation of HR activity. More *cku-80* animals expressed GFP than wild types (Figure 6D). This increase was likely an underestimation of HR activity as the GFP was also expressed much more brightly in *cku-80* mutants than wild types. Brighter GFP likely results from multiple HR reporter genes being activated within a single cell. These experiments demonstrated that the HR reporter is able to measure relative changes in HR activity, in both HR-deficient and HR-hyperactive mutants.

Importantly, we found that both *lin-61(n3809)* and *lin-61(pk2225)* mutants showed a substantial reduction in the frequency of HR reporter activation compared with wild types (Figure 6D). In fact HR activation in *lin-61* mutants was reduced to *brc-1* levels. This confirmed LIN-61 is needed for DSB repair by the HR pathway. Further, it indicated that IR hypersensitivity of *lin-61* mutants was likely due to defective DSB repair rather than other types of IR-induced cellular damage. While HR repairs DSBs in an error-free way, other DSB repair pathways such as NHEJ and SSA are error-prone processes. To test whether LIN-61 contributes to mutagenic DSB repair routes, we constructed a second reporter gene that specifically monitored SSA. This SSA reporter was similar to the HR reporter as both were expressed in intestinal nuclei and both received a single DSB from the mCherry::I-SceI enzyme, however the SSA reporter could only become active following an SSA event, and not an HR event (Figure S6A). We found that *lin-61* mutants did not have reduced SSA activity but actually had increased SSA reporter activation compared to wild types (Figure S6B–S6C), in line with *lin-61* mutants being HR-defective. A similar shift towards SSA has previously been found for DSB repair in *brc-1* mutant animals [41]. We conclude that LIN-61 is necessary for efficient HR in somatic cells but is dispensable for SSA in somatic intestinal cells. Assays that measure sensitivity to DNA-damaging agents revealed that embryonic and germline cells of *lin-61* mutants are defective for DSB repair (Figure 2 and Figure 3). The data generated using the HR and SSA reporters demonstrated that cell types other than those of the germline and embryo are defective for DSB repair in *lin-61* mutants. Together, these complementary experiments suggested that *lin-61* mutants have a systemic defect in DSB repair.

### DNA damage checkpoints are proficient in *lin-61* mutants

**Figure 7:**
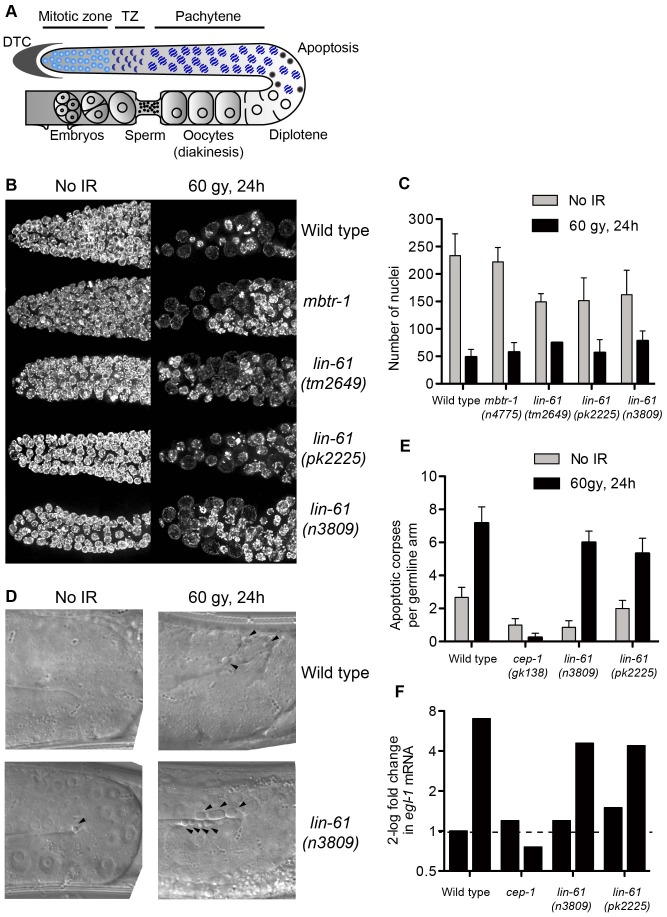
LIN-61 is dispensable for DNA damage checkpoints in the germline. (A) Schematic diagram of the hermaphrodite germline. Cell cycle arrest (as in B–C) occurs in the mitotic zone and apoptosis (D–E) occurs at the bend of the germline. DTC, distal tip cell; TZ, transition zone. (B) Maximum projections of DAPI-stained mitotic nuclei 24 hours after irradiation with 60 Gy or mock-treatment. (C) Quantification of mitotic cell cycle arrest, error bars are s.d. (D) DIC images of pachytene stage nuclei 24 hours after irradiation with 60 Gy or mock-treatment. Arrowheads mark apoptotic corpses. (E) Quantification of apoptotic corpses per germline arm. Error bars represent s.d. (F) Quantification of *egl-1* mRNA by qRT-PCR, normalised to untreated wild types. Total RNA was isolated from mixed populations of developmentally staged young adults 24 hours after irradiation with 120 Gy, or mock treatment.

Sensitivity to DNA damage can be caused by failure to activate DNA damage checkpoints [43]. The G2/M checkpoint is triggered in response to DNA damage and keeps mitotic germ cells in G2 phase to provide sufficient time for DNA repair (Figure 7A) [44]. Arrested cells do not divide, but continue to grow, making them readily identifiable by their enlarged size [43]. Following exposure to IR, all three *lin-61* mutants displayed proficient cell cycle arrest. Like wild type worms (and *mbtr-*1 mutants that are not IR sensitive), the *lin-61* mutants had enlarged mitotic nuclei and a reduced number of germ cells 24 hours after γ-irradiation (Figure 7B–7C).

In addition to the G2/M checkpoint, DNA damage also triggers apoptosis in pachytene stage meiotic cells via a process dependent upon the p53 homologue, CEP-1 [43], [45]. Upon challenge with IR, apoptotic corpses accumulated in the germlines of wild type, *lin-61(n3809)* and *lin-61(pk2225)* animals, while *cep-1* mutants failed to undergo DNA damage-dependent apoptosis (Figure 6D–6E). CEP-1 drives the apoptotic programme by up-regulating *egl-1*/BH3-only transcription [43], [45], [46]. In response to IR, *egl-1* expression was increased in wild type and *lin-61* worms, but not *cep-1* mutants, as determined by qRT-PCR (Figure 7F). Together these results indicated that the activation of DNA damage checkpoints (cell cycle arrest and apoptosis) was normal in *lin-61* mutants. The hypersensitivity of *lin-61* mutants to IR could therefore not be attributed to defective checkpoint activation.

### DNA repair genes are expressed at normal levels in *lin-61* mutants

Since LIN-61 is a transcriptional repressor, we checked whether DDR genes were appropriately expressed in *lin-61* mutants, as this could be the underlying cause of their HR defect. Using microarrays, we compared the expression profiles of wild types and *lin-61* animals. Young adult worms (24 hours post L4) were analysed in order to increase the proportion of germ cells present in the samples, considering LIN-61 is needed for repair of DSBs in both somatic and germ cells. Microarrays were performed on two different *lin-61* alleles *(n3809* and *pk2225)* in order to control for changes in gene expression that were due by background mutations present within only one of the single strains. 58 genes were identified that, in both mutants, had a 1.5-fold or greater change in expression level (p-value<0.01) (Table S1). Most of these alternatively expressed genes were upregulated in *lin-61* mutants (52 genes, 90%), with only 6 genes (10%) downregulated. This is consistent with LIN-61 acting as a transcriptional repressor. Importantly, none of the genes alternatively expressed in *lin-61* mutants were implicated in DNA repair. The *lin-61* transcript served as a positive control in the microarray analysis as we had previously shown, using qRT-PCR, that this transcript was reduced approximately 4-fold in *lin-61* mutants, likely due to nonsense-mediated decay (Figure 1B). According to the microarray data, *lin-61* mRNA was reduced 3.25-fold, which in good agreement with the qRT-PCR data. The expression analysis showed that while LIN-61 does indeed act as a transcriptional repressor, *lin-61* mutation by itself (in the absence of an additional synMuvA mutation) has only a minor effect on global gene transcription. Finally, since these experiments indicated that DNA repair genes are expressed at normal levels in *lin-61* mutants, it is likely that LIN-61 influences DSB repair directly and not by ensuring that other DDR genes are appropriately expressed.

## Discussion

In this study we have identified the underlying cause of genomic instability in *lin-61* mutants: DSBs are not adequately repaired due to defective HR. Accordingly, these animals are hypersensitive to IR and nitrogen mustard and DSBs remain unrepaired in diakinesis oocytes of γ-irradiated *lin-61* mutants. LIN-61 contributes to HR in mitotic cells but it is dispensable for DSB repair during meiosis. Sensitivity of *lin-61* germ cells to DSBs is not due to faulty DNA damage checkpoints as both cell cycle arrest and apoptosis are functional. Moreover, DNA repair genes are not inappropriately expressed in *lin-61* mutants. The role of LIN-61 in HR is not restricted to germ cells because the somatic cells of early stage embryo are also very sensitive to IR. Also, later in development, intestinal cells are HR defective, as determined by the GFP-based HR reporter system. HR is essential for genome stability, as it is the principal DSB repair route in germ cells. It is also an error-free repair pathway. Blocking HR enables mutagenic and toxic repair routes to become active, which likely contributes to genomic instability in *lin-61* mutants.

### The role of LIN-61 in HR is restricted to mitotic cells

LIN-61 is expressed in all nuclei, both in the germline and somatic tissues [6]. Despite this, several observations suggests that LIN-61 contributes to HR only in mitotic cells and is dispensable for both meiotic interhomolog and intersister HR. Meiotic cells rely on interhomolog HR to repair at least one programmed DSBs per chromosome pair so that the obligate CO will be established [47]. Meiotic recombination is not defective in *lin-61* mutants as they form chiasmata normally and produce nearly completely viable broods. What is more, RAD-51 foci that appear in prophase are resolved by late pachytene in both wild type and *lin-61* mutants, indicative of the successful repair of programmed DSBs. The proficiency of intersister HR can be tested in meiotic cells by disrupting the SC in order to prevent interhomolog HR. In this situation, DSBs remain unrepaired if intersister HR too is defective, which manifests as chromosomal fragmentation at diakinesis. Unlike *brc-1* and *smc-5/-6* mutants [19], [27,27], *lin-61* mutants depleted of the SC component SYP-2 do not have fragmented diakinesis chromosomes, indicating that intersister HR is proficient in the meiotic cells of these mutants. Moreover, DSBs introduced by IR into *lin-61* meiotic cells, but not *brc-1* meiotic cells, are efficiently repaired.

While *lin-61* mutants are proficient in meiotic HR, their mitotic cells are defective in HR. These cells display signs of persistent and spontaneous DNA damage. Further, γ-irradiation of mitotic germ cells causes severe chromosome fragmentation in *lin-61* mutants. Finally, *lin-61* mutants are also hypersensitive to ICLs and the repair of these lesions occurs in S/G2 phase using the newly synthesised sister chromatid as the HR repair template [29]. The somatic (mitotic) cells of *lin-61* are also hypersensitive to IR and mitotic cells exclusively use the sister chromatid for HR [39]. Together, these observations indicate that LIN-61 contributes to DSB repair via intersister HR in mitotic cells but does not participate in meiotic HR.

### How does LIN-61 promote DSB repair?

Considering that the transcriptional profile of *lin-61* mutants cannot explain their HR defect, LIN-61 likely acts directly at sites of DNA damage to promote DSB repair. This is an attractive hypothesis considering that chromatin can act as a physical barrier that must be remodelled to allow access of DDR factors to sites of damage. In addition, many proteins that alter chromatin structure have recently been implicated in the DDR including NuRD components MTA1, MTA2, CHD4, HDAC1 and HDAC2 [48]–[50]; and PcG proteins BMI1, RING1, RING2 and HP1 [51]–[55]. Each of these proteins is rapidly recruited to DNA damage and is necessary for DNA repair. The *C. elegans* counterparts of these proteins are also synMuvB proteins like LIN-61. Intriguingly, L3MBTL2, the putative human orthologue of LIN-61, is part of a PcG-like complex (PRC1L4) that shares RING1, RING2 and HP1γ as partner members [56]. Moreover, human cells depleted of RING2 [55], and *C. elegans hlp-2* HP1 mutants [53], are radiosensitive like *lin-61* mutants. PRC1L4, or a related L3MBTL2-containing PcG complex, may therefore act in DSB repair like LIN-61. Using immunofluorescence, we were not able to detect a change in LIN-61 intracellular localisation upon IR (data not shown). However LIN-61 is abundantly present and localised at chromatin in all cells, which may conceal its relocalisation around sites of DNA damage. Recruitment to sites of DNA damage has also not been observed for any other *C. elegans* synMuvB proteins, likely for similar reasons.

It is unknown how PcG activity promotes DSB repair but it is argued that inhibiting transcription locally at the DSB may be important as the transcriptional machinery could interfere with repair proteins or with DNA repair intermediates [50], [57]. PRC1L4 represses transcription of target genes by monoubiquitinating lysine 119 of histone H2A via its E3 ubiquitin ligase activity [56]. This histone mark is also implicated in the DDR as it was recently shown to rapidly accumulate at DSBs [52], [58]. It will be of interest to determine whether L3MBTL2 and the other members of PRC1L4 are involved in DSB repair in human cells.

One possible explanation we considered for why *lin-61* mutants were HR-defective was that they might have altered expression of DDR genes. But contrary to this, microarray expression analysis did not reveal any alternatively expressed DDR genes in these mutants. Some alternatively expressed genes were identified but none are implicated in DNA repair. The vast majority of the alternatively expressed genes were upregulated rather than downregulated, which is in accordance with LIN-61 being a transcriptional repressor. A previous study found that germline genes were ectopically expressed in the somatic tissues of *lin-61* mutants, but only when maintained at the relatively high temperature of 26°C [13]. In line with this, we found that *lin-61* mutants grown at the normal laboratory temperature of 20°C had only minor changes in gene expression and did not overexpress germline genes. Importantly, *lin-61* mutants grown at 20°C displayed a profound HR defect, which further indicated that altered gene expression was not the cause of defective DNA repair. The microarrays were performed using RNA from a mixed population of germ and somatic cells. We cannot strictly exclude the possibility that a distinct population of cells had altered DDR gene expression that went undetected. This is unlikely though, as the defect in DSB repair was systemic, occurring in multiple tissues and at various stages of development, and not isolated to a small number of cells.

### A novel GFP-based HR reporter system for *C. elegans*

In this study we introduce a novel reporter system for monitoring HR in *C. elegans* somatic cells. The reporter confirmed that LIN-61 is needed for HR. This tool was previously unavailable for *C. elegans* researchers. We propose it as a method for testing candidate HR genes, for example it confirmed that both BRC-1 and RTEL-1 have roles in HR in somatic cells, analogous to their functions previously only described in meiotic germ cells. Our experiments with the HR reporter also supported previous findings that suggested DSB repair pathways are dynamic and are in competition in somatic cells [41] since mutations that blocked NHEJ, increased HR reporter activity.

Though this system is a new tool that provides for the readout of repair, probably by an SDSA mechanism, of a defined DSB, it does have limitations. For example, the HR reporter does not easily allow for dissection of the biochemical processes that underpin HR pathways. These approaches are not well suited to *C. elegans*. Also, in its current form the HR reporter is expressed only in intestinal cells, which in contrast to most *C. elegans* somatic cells still cycle postembryoniccally. This choice of cell type was largely motivated by the likely need for S- and G2 phase dependent DNA end resection at DSBs for HR type of repair to occur. However, when interpreting the data it must be considered that these cells are atypical because they progress and grow through cycles of endoreduplication and not via canonical cell cycle stages including mitosis. It is thus possible that the response to the HR reporter is cell type-dependent. Finally, since formation of the DSB relies on expression of the I-SceI transgene using the heatshock promoter, any possible differences in heatshock response must be carefully controlled for as these differences may affect the level of DSB induction.

### Implications for HR deficiency in MBT mutants

This is the first report showing that an MBT protein is needed for DSB repair. Genes encoding MBT proteins have previously been linked with tumourigenesis and can act as tumour suppressor genes. However, their contribution to DNA repair and genome stability is unknown. Our finding that LIN-61 is required for efficient HR may have implications for the treatment of MBT-deficient tumours, which may also be HR defective. HR-deficient tumours, such as those with BRCA1 or 2 hypomorphic mutations, are very susceptible to poly(ADP ribose) polymerase (PARP) inhibitors [39]. It will be important to determine whether the role of LIN-61 in DSB repair is conserved in human MBT proteins and whether MBT mutated tumours, such as medulloblastomas with mutations in *L3MBTL2*, *L3MBTL3* or *SCML2* [10], are HR deficient as they too may prove responsive to treatment with PARP inhibitors.

## Materials and Methods

### Genetics

The Bristol N2 strain was used as the wild type strain and maintained at 20°C according to standard protocols [59]. Alleles used in the study include LG I: *lin-61(n3809)* [6], *lin-61(pk2225)* (this study), *lin-61(tm2649)* [15], *mbtr-1(n4775)* [6], *cep-1(gk138)* [60] and *rtel-1(tm1866)* [61]; LG III: *brc-1(tm1145)* [62], *cku-80(ok861)* [63], *polh-1(lf31)* [31], *lfIs129 [elt-2::HR-reporter; hsp16-41::mCherry::I-SceI]* (this study); and LG X: *lfIs82 [elt-2::SSA-reporter; hsp16-41::mCherry::I-SceI]* (this study). To determine brood sizes, L4 larvae were singled on 6 cm plates with OP50 *E. coli* and transferred each day for three days. The number of viable progeny and unhatched eggs were counted, as well as the number of males in the brood.

### DNA damage sensitivity, checkpoint activation, and chromosome fragmentation assays

All γ-irradiation was performed with a dose rate of 15 Gy/minute using an electronic X-ray generator set to 200 kV 12 mA (XYLON International). For L4 larval IR sensitivity, three L4 animals per plate (three plates per condition) were treated with various doses of γ-irradiation. For UV-C sensitivity, young adult (24 post L4 stage) worms were exposed to UV (254 nm lamp, Philips). HN2 sensitivity assays were performed as described [64]. γ-irradiation of embryos and L1 larvae was preformed as described [32]. Apoptosis assays were performed in as [45]. Cell cycle arrest and fragmentation assays were as in [36]. *syp-2* RNAi was performed as in [19]. For cell cycle arrest, 4–5 germlines were analysed per condition, except for irradiated *lin-61(tm649)* for which a single germ line was scored.

### Germline dissections and RAD-51 immunofluorescence

Germlines were dissected in egg salts, Tween, levamisole and fixed in 2% paraformaldehyde for 5 minutes at room temperature, and snap frozen on dry ice, then placed in methanol at −20°C for 10 minutes, washed three times for 10 minutes in PBS with 1% Triton X-100 and blocked in PBST (PBS with 0.1% Tween 20) and 1% BSA for 30 minutes at room temperature. Samples were incubated overnight at 4°C with rabbit anti-RAD-51 antibodies (Novus Biologicals) diluted 1∶200 in PBST 1% BSA and detected with Alexa488 goat anti-rabbit antibodies (Invitrogen) diluted 1∶1000. DNA was counterstained with 0.5 µg/ml DAPI and samples were mounted with VectaShield. RAD-51 foci were imaged with a Leica DM6000 deconvolution microscope collecting 0.5 µm Z-sections. The number of foci per nucleus was counted for each of the seven zones of the germline as described [64]. Three to five germlines were quantified per condition.

### Microarray and qRT–PCR

Worms were synchronised as L1 larvae by bleaching and grown to the L4 stage. Total RNA was isolated with Trizol reagent (Invitrogen), and cleaned with RNeasy kit (Qiagen). Service XS (Leiden, NL) performed the Affymetrix expression analysis according to standard protocols. Data was analysed with the MAS 5.0 algorithm using Tukey's biweight estimator. Significance (p-value) was determined using Wilcoxon's rank test. Sequence of qRT-PCR primers is available in Text S1.

### P*elt-2*::HR and P*elt-2*::SSA reporter

Details on construction of the P*elt-2*::HR and P*elt-2*::SSA reporter strains are provided in Text S1. For HR reporter assays, expression of mCherry::ISce-I was induced in L4 larvae by heatshock twice at 34°C for 1 hour (with 30 min rest at 20°C). 24 hours after induction, worms were mounted on agarose pads and their intestinal nuclei were scored for GFP expression using a Leica DM6000 microscope with 63× objective. Experiments were performed in triplicate with 50–100 animals tested for each condition. Statistical significance was tested using the Cochran-Mantel-Haenszel test.

## Supporting Information

Figure S1 The primordial germ cells of *lin-61* mutants are hypersensitive to IR. L1 larvae were irradiated with the indicated dose of IR and grown to adulthood before their brood sizes was determined. The average brood size of five adults was counted for each condition. Depicted is the average brood size from two experiments, normalised to the brood size of unirradiated animals. Error bars are standard error of the mean.
(JPG)

Figure S2 mbtr-1 mutants are not sensitive to IR. (A) Protein sequence alignment of LIN-61 and MBTR-1. Asterisk (*), semicolon (:) and full stop (.) denote identical residues, conserved substitutions and semi-conservative substitutions, respectively. Residues present in the four MBT domains are coloured red, blue, green and purple. (B) *mbtr-1* mutants are not sensitive to IR. The percentage of viable progeny laid by irradiated L4 larvae is plotted. Error bars represent standard deviation.
(PDF)

Figure S3 Quantification of RAD-51 foci in lin-61 germlines. (A) Stacked histograms showing the average number of RAD-51 foci per nucleus present in each of the seven zones of the germline. (B) Diagram depicting the germline divided into seven zones. Zones one and two include the mitotic zone; zone three is the transition zone (TZ); zones four and five are early-mid pachytene; zone six is late pachytene; and zone seven is late pachytene/diplotene. DTC, distal tip cell.
(PDF)

Figure S4 *brc-1* L1 larvae do not display developmental delay following IR. Depicted is the proportion of animals that developed to the L4 stage 48 hours after being γ-irradiated as L1 larvae with the indicated dose. Error bars are s.d.
(PDF)

Figure S5 HR reporter activation requires donor sequence for activation. (A) Schematic diagram of versions of the HR reporter that contain (upper panel; strain XF460) or lack (lower panel; strain XF444) the gfp donor cassette. These reporters are expressed using the heatshock promoter. (B) Epifluorescence and brightfield images of adult worms 24 hours after DSB induction. GFP is visible in intestinal cells in XF460, but not XF444.
(PDF)

Figure S6 Pelt-2::SSA reporter. (A) Schematic showing the Pelt-2::SSA reporter. The Pelt-2::SSA reporter consists of an out-of-frame LacZ gene, disrupted by an I-SceI sites and stop codons in all three frames. A region of LacZ is duplicated and located between the elt-2 promoter and the I-SceI site, and provides homologous sequences for SSA. A DSB is introduced in the centre of the reporter by expressing Pheatshock::mCherry::I-SceI. Repair of the DSB by SSA places the LacZ gene in-frame and deletes the sequences between the homologous repeats (including the I-SceI site and stop codons). (B) LacZ (β-galactosidase) activity was visualised by the conversion of X-gal to 5,5′-dibromo-4,4′-dichloro-indigo, which has an intense blue colour. Shown are representative bright field images of L4/young adult worms expressing LacZ in their intestinal cells (C) Graph showing the percentage of worms containing at least one blue intestinal cell. Induction of mCherry::I-SceI was achieved by heatshocking L1 stage worms for 30 or 60 min. These worms were stained for LacZ expression 48 hours after heatshock. Error bars represent standard deviation.
(JPG)

Table S1 Genes alternatively expressed in *lin-61* mutants. This table lists the genes alternatively expressed in L4 stage *lin-61* mutants compared with wild types, as determined by Affymetrix expression analysis.
(XLSX)

Text S1 Supporting Experimental Procedures. Methods and materials are described for the following supporting experimental procedures: Description of *lin-61* mutant alleles; construction of P*elt-2*::HR reporter, P*elt-2*::SSA reporter and P*hsp16-41*::mCherry::ISce-I; SSA reporter assay; list of qRT-PCR primers and L1 larvae IR assay.
(DOCX)
