# Supplementary figures and images for: A Role for the Malignant Brain Tumour (MBT) Domain Protein LIN-61 in DNA Double-Strand Break Repair by Homologous Recombination
Source: PLoS Genet. 2013 Mar 7;9(3):e1003339. doi: 10.1371/journal.pgen.1003339 (PMC3591299; doi:10.1371/journal.pgen.1003339)

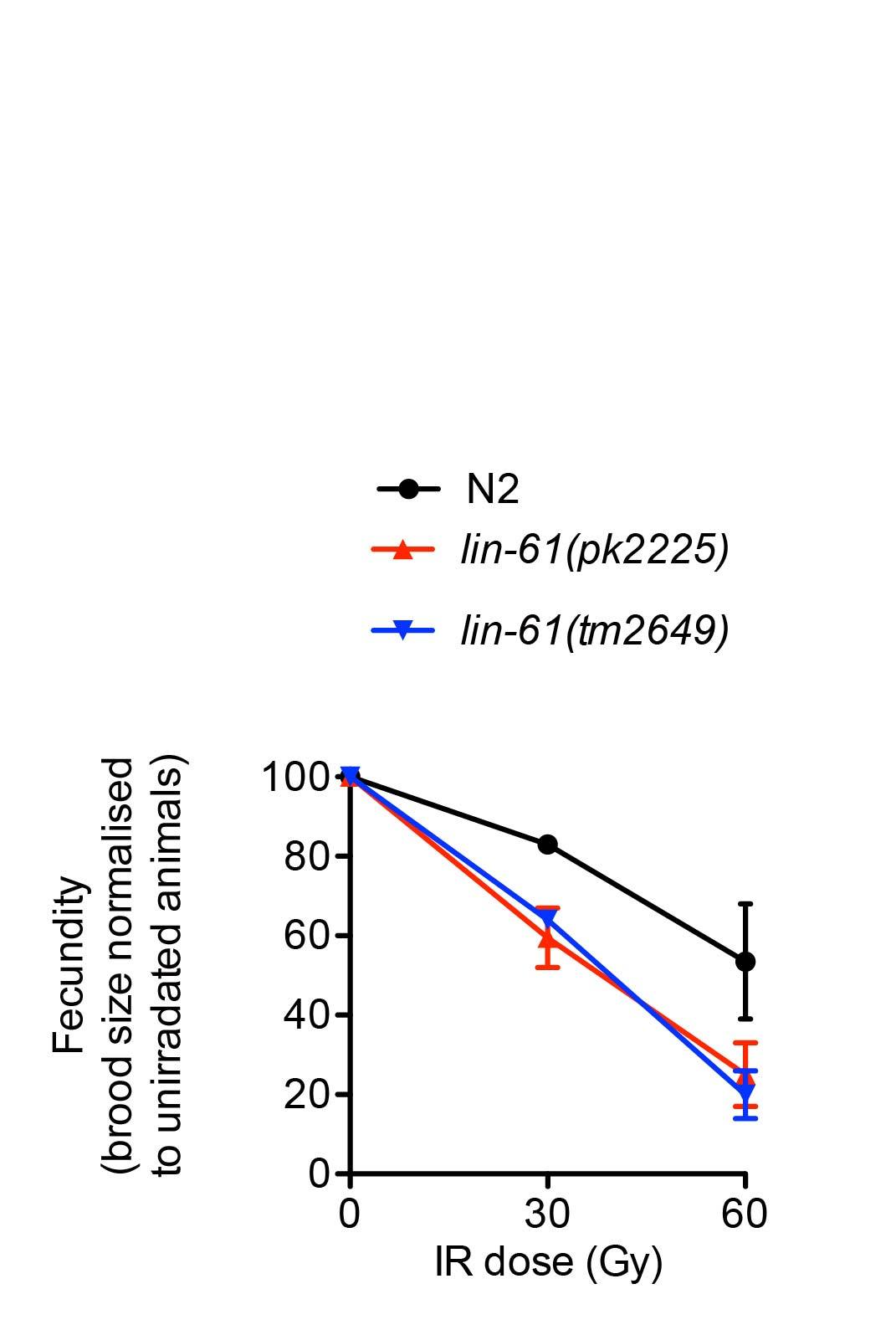

Supplement: Figure S1 — The primordial germ cells of lin-61 mutants are hypersensitive to IR. L1 larvae were irradiated with the indicated dose of IR and grown to adulthood before their brood sizes was determined. The average brood size of five adults was counted for each condition. Depicted is the average brood size from two experiments, normalised to the brood size of unirradiated animals. Error bars are standard error of the mean. (JPG) [file pgen.1003339.s001.jpg]

**A**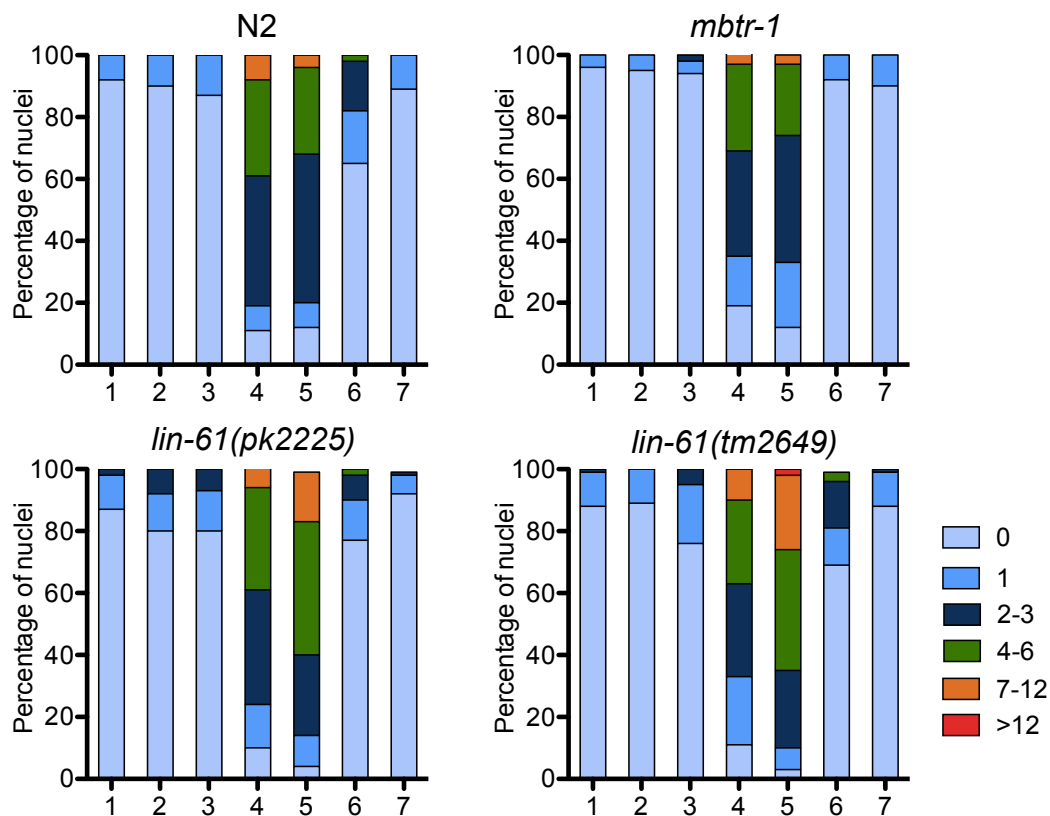**B**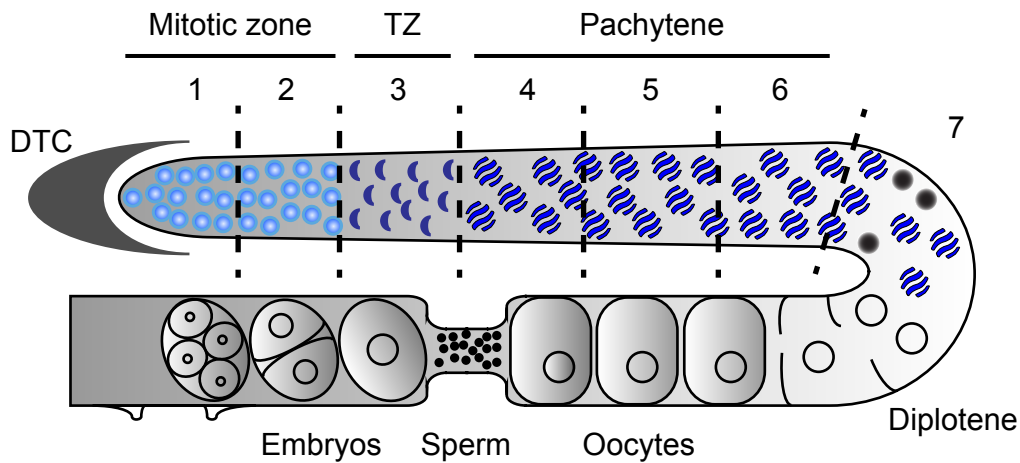

Supplement: Figure S3 — Quantification of RAD-51 foci in lin-61 germlines. (A) Stacked histograms showing the average number of RAD-51 foci per nucleus present in each of the seven zones of the germline. (B) Diagram depicting the germline divided into seven zones. Zones one and two include the mitotic zone; zone three is the transition zone (TZ); zones four and five are early-mid pachytene; zone six is late pachytene; and zone seven is late pachytene/diplotene. DTC, distal tip cell. (PDF) [file pgen.1003339.s003.pdf]

■ Wild type ■ *brc-1* ■ *cku-80*

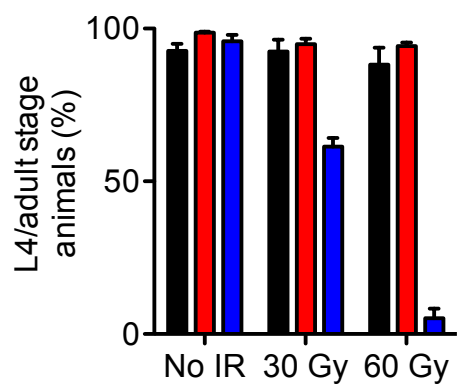

Supplement: Figure S4 — brc-1 L1 larvae do not display developmental delay following IR. Depicted is the proportion of animals that developed to the L4 stage 48 hours after being γ-irradiated as L1 larvae with the indicated dose. Error bars are s.d. (PDF) [file pgen.1003339.s004.pdf]

**A**XF460 (HR reporter with donor *gfp* sequences)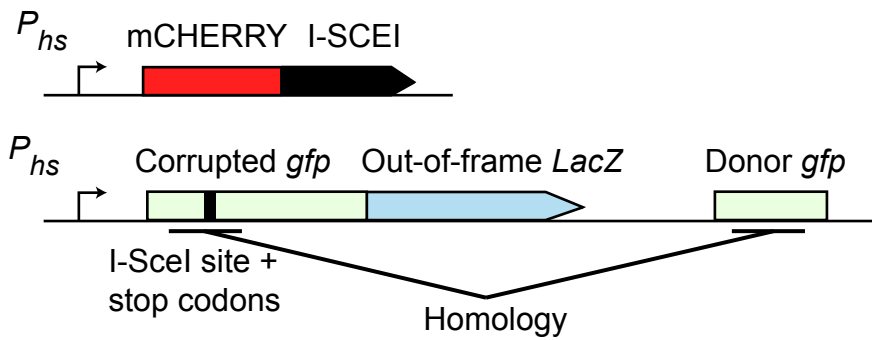XF444 (HR reporter with donor *gfp* sequences deleted)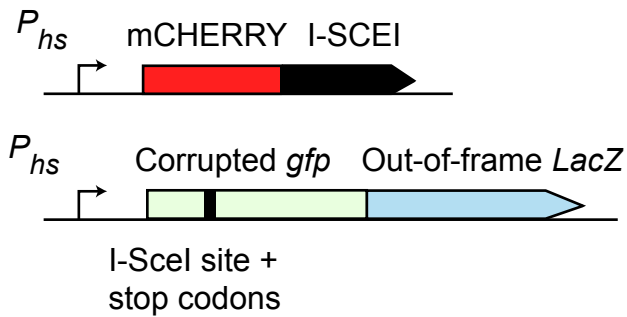**B**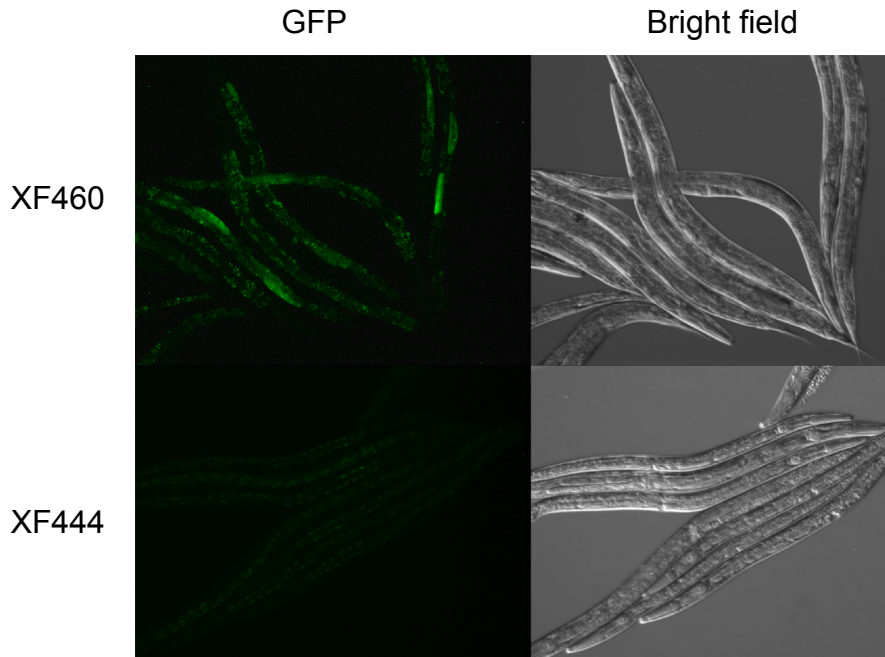

Supplement: Figure S5 — HR reporter activation requires donor sequence for activation. (A) Schematic diagram of versions of the HR reporter that contain (upper panel; strain XF460) or lack (lower panel; strain XF444) the gfp donor cassette. These reporters are expressed using the heatshock promoter. (B) Epifluorescence and brightfield images of adult worms 24 hours after DSB induction. GFP is visible in intestinal cells in XF460, but not XF444. (PDF) [file pgen.1003339.s005.pdf]

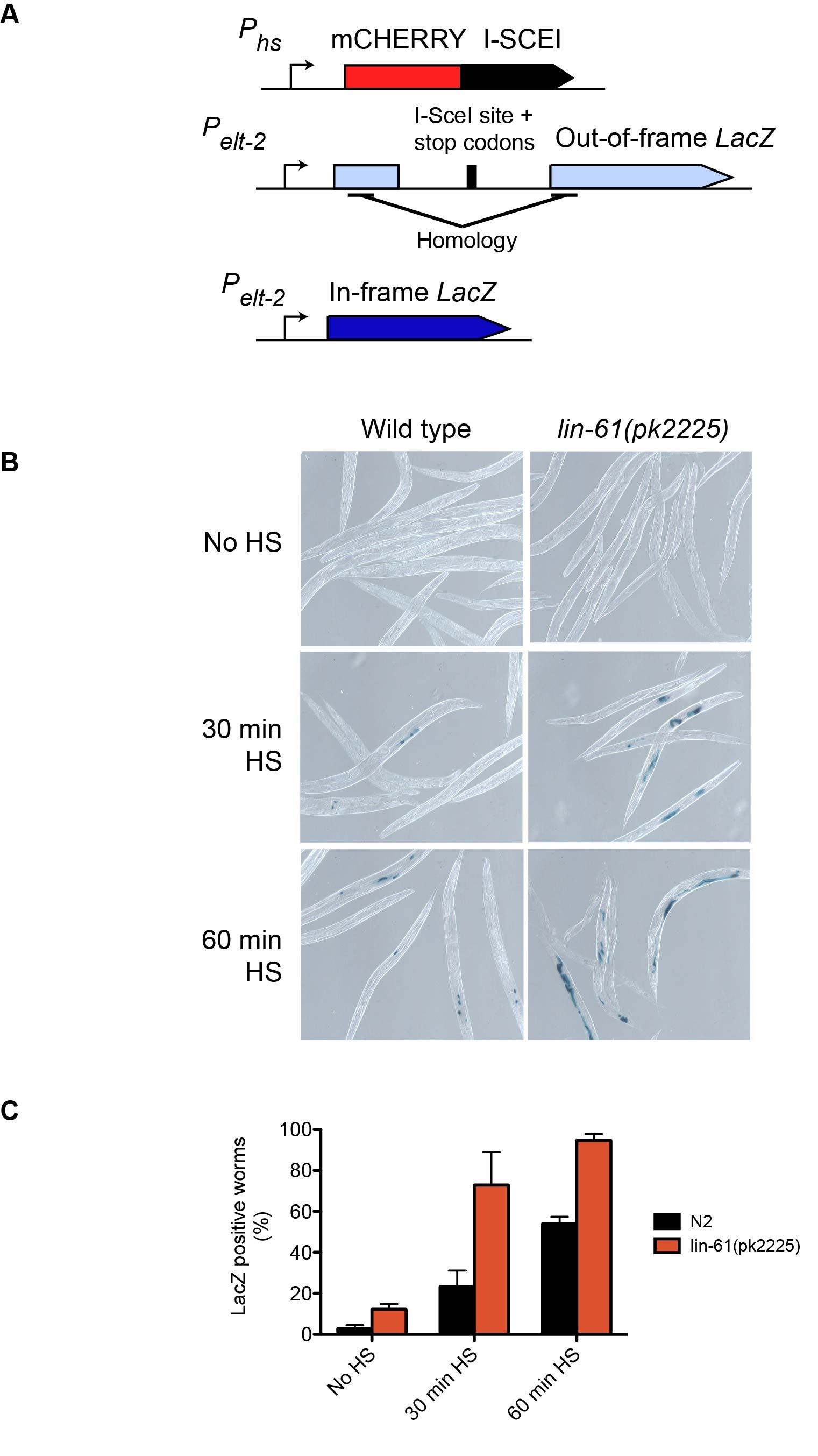

Supplement: Figure S6 — Pelt-2::SSA reporter. (A) Schematic showing the Pelt-2::SSA reporter. The Pelt-2::SSA reporter consists of an out-of-frame LacZ gene, disrupted by an I-SceI sites and stop codons in all three frames. A region of LacZ is duplicated and located between the elt-2 promoter and the I-SceI site, and provides homologous sequences for SSA. A DSB is introduced in the centre of the reporter by expressing Pheatshock::mCherry::I-SceI. Repair of the DSB by SSA places the LacZ gene in-frame and deletes the sequences between the homologous repeats (including the I-SceI site and stop codons). (B) LacZ (β-galactosidase) activity was visualised by the conversion of X-gal to 5,5′-dibromo-4,4′-dichloro-indigo, which has an intense blue colour. Shown are representative bright field images of L4/young adult worms expressing LacZ in their intestinal cells (C) Graph showing the percentage of worms containing at least one blue intestinal cell. Induction of mCherry::I-SceI was achieved by heatshocking L1 stage worms for 30 or 60 min. These worms were stained for LacZ expression 48 hours after heatshock. Error bars represent standard deviation. (JPG) [file pgen.1003339.s006.jpg]
