# Supplementary material for: A Role for the Malignant Brain Tumour (MBT) Domain Protein LIN-61 in DNA Double-Strand Break Repair by Homologous Recombination
Source: PLoS Genet. 2013 Mar 7;9(3):e1003339. doi: 10.1371/journal.pgen.1003339 (PMC3591299; doi:10.1371/journal.pgen.1003339)

A

```
MBTR-1 ------------------------------------------------------------
LIN-61 MLKLVILCFALFYNTVSSTRFLFGVEVKCDFDEVFQLTVSHWEDDGNTFWDRDEDITGRM 60

MBTR-1 --MEKSSSNFIQNGKR-----------------DHGGKLRQYNYKLEEAEYRYFTEERLF 41
LIN-61 TMFARKKIFFYQDGHHGFEFGKLEPYGWFLHNCTKNGNFREYRHGLSSTSGSNGLEYIEY 120

MBTR-1 YRRRNPVEKIAQRIPKPQIEGTFTWSDELRCNYDGNTQFLPVEALEGCLPLEKLNQHLKP 101
LIN-61 TMSEFLKIVRANKKSDRKLDKTYLWESYLHQFEKGKTSFIPVEAFNRNLTVN-FNECVKE 179
          .      *:: .. ::: *: *.. *:   .*:*.*:****::  *.:: :*:  :*
MBTR-1 GFRLEVVVRPSLDPSITTKSPEIRWFGEVTAVCGFYVAIKFVGELNRRPCWFHMLSEDIF 161
LIN-61 GVIFETVVH---DYDKNCDSIQVRWFARIEKVCGYRVLAQFIGADTK--FWLNILSDDMF 234
       *. :*.**:   * . . .* ::***..:  ***: *  :*:*  .:   *:::**:*:*
MBTR-1 DIGSGLKQDPAMKWLQYR-PLSLLKPMQCPKFWRRGSTPAPPVPRPTEEILDEFQAELHE 220
LIN-61 GLANAAMSDPNMDKIVYAPPLAINEEYQN---------------DMVNYVNNCIDGEIVG 279
       .:...  .** *. : *  **:: :  *                  .: : : ::.*:
MBTR-1 NRISEPKIFDQLRHLAHRPSRFRLNQRVELLNYLEPTEIRVARILRILGRRLMVMVTAQD 280
LIN-61 -QTSLSPKFDEGKALLSK-HRFKVGQRLELLNYSNSTEIRVARIQEICGRRMNVSITKKD 337
        : * .  **: : *  :  **::.**:***** :.******** .* ***: * :* :*
MBTR-1 YPEDLPSVEAKDRQVQHENVEFWVDESSFFLFPVGFAMINGLRTKATEGYLEHSRRIAEG 340
LIN-61 FPESLPDADD-DRQVFSSGSQYWIDEGSFFIFPVGFAAVNGYQLNAKKEYIEHTNKIAQA 396
       :**.**..:  ****  .. ::*:**.***:****** :** : :*.: *:**:.:**:.
MBTR-1 SGS-----YHKDDVTFEQLFAGKPDISAEKLNLLKVGQKFELLDPLSDLRQSFCVATIRK 395
LIN-61 IKNGENPRYDSDDVTFDQLAKDP--IDPMIWRKVKVGQKFELIDPLAQQFNNLHVASILK 454
         .     *..*****:**  .   *..   . :********:***::  :.: **:* *
MBTR-1 ICKTPGFLIISPDETESDDESFPIHIDNHFMHPVGYAEKFGIKLDRLAGTEPGKFKWEGY 455
LIN-61 FCKTEGYLIVGMDGPDALEDSFPIHINNTFMFPVGYAEKYNLELVPPD-EFKGTFRWDEY 513
       :*** *:**:. * .:: ::******:* **.*******:.::*        *.*:*: *
MBTR-1 LKEKQAEKIPDEMLRPLPSKERRHMFEFGRVLEAVGQNETYWISPASVEEVHGRTVLIEF 515
LIN-61 LEKESAETLPLDLFKPMPSQERLDKFKVGLRLEAADMCENQFICPATVKSVHGRLINVNF 573
       *:::.**.:* ::::*:**:** . *:.*  ***..  *. :*.**:*:.**** : ::*
MBTR-1 QGWDSEFSELYDMDSHDLLPAGWCEFFNFKLRHPVLPVNDPNAENGEYD 564
LIN-61 DGWDEEFDELYDVDSHDILPIGWCEAHSYVLQP-------PKKYN--Y- 612
       :***.**.****:****:** **** ..: *:        *:  *  *
```

B

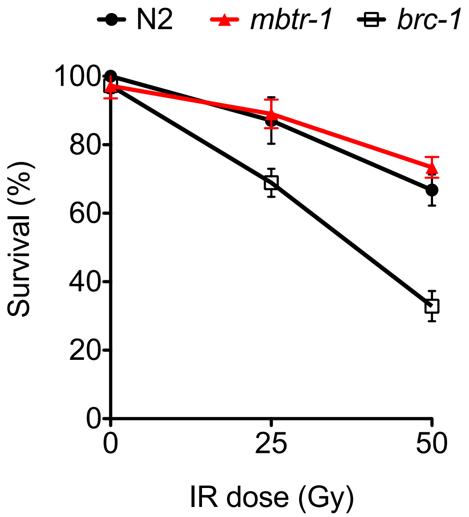

Supplement: Figure S2 — mbtr-1 mutants are not sensitive to IR. (A) Protein sequence alignment of LIN-61 and MBTR-1. Asterisk (*), semicolon (:) and full stop (.) denote identical residues, conserved substitutions and semi-conservative substitutions, respectively. Residues present in the four MBT domains are coloured red, blue, green and purple. (B) mbtr-1 mutants are not sensitive to IR. The percentage of viable progeny laid by irradiated L4 larvae is plotted. Error bars represent standard deviation. (PDF) [file pgen.1003339.s002.pdf]
