# Supplementary material for: A Role for the Malignant Brain Tumour (MBT) Domain Protein LIN-61 in DNA Double-Strand Break Repair by Homologous Recombination
Source: PLoS Genet. 2013 Mar 7;9(3):e1003339. doi: 10.1371/journal.pgen.1003339 (PMC3591299; doi:10.1371/journal.pgen.1003339)
Supplement: Text S1 — Supporting Experimental Procedures. Methods and materials are described for the following supporting experimental procedures: Description of lin-61 mutant alleles; construction of Pelt-2::HR reporter, Pelt-2::SSA reporter and Phsp16-41::mCherry::ISce-I; SSA reporter assay; list of qRT-PCR primers and L1 larvae IR assay. (DOCX) [file pgen.1003339.s008.docx]

**Text S1**

**Supporting Experimental Procedures**

**Description of *lin-61* mutant alleles**

*n3809* is a CAA to TAA nonsense mutation (Q159ochre) located in the fourth exon that truncates the LIN-61 protein by removing the second, third and fourth MBT domains and causes a highly penetrant synMuv phenotype (Harrison et al., 2007) The second allele, *pk2225,* was isolated from an EMS mutagenesis library (Cuppen et al., 2007) and identified using a reverse genetics approach by sequencing PCR amplicons of the *lin-61* gene. *pk2225* is also a CAA to TAA mutation (Q412ochre) and, coincidently, is identical to another allele (*n3446*) isolated in a screen for mutants that have a synMuv phenotype when combined with *lin-15A* (Ceol et al., 2006). The synMuv phenotype of *n3446* was as penetrant as *n3809* (Harrison et al., 2007), therefore *pk2225* is also likely to have a strong synMuv phenotype. The third allele (*tm2649*) is a large deletion within *lin-61* with breakage points located in the second exon and at a position in the fourth exon that places the remaining exons out-of-frame (Figure 1A). *lin-61(tm2649)* also causes a highly penetrant synMuv phenotype (Koester-Eiserfunke and Fischle, 2011).

**Construction of P*elt-2*::HR reporter, P*elt-2*::SSA reporter and P*hsp16-41*::mCherry::ISce-I**

pLM44 (P*elt-2*::SSA reporter) was constructed by transferring the cassette containing LacZ (interrupted by an I-SceI site) from pRP1879 (Pontier and Tijsterman, 2009) to pJM67 (P*elt-2*::gfp::lacZ) using AgeI and XhoI sites. pLM17 (P*hsp16-41*::mCherry::ISceI) was constructed by inserting an mCherry cassette into pRP3001 (P*hsp16-41*::ISceI) using a single XmaI site to produce an in-frame N-terminal fusion. The plasmids were injected together at 2 ng/µl along with pRF4 (dominant *rol-6(su1006)* marker) and genomic DNA to generate transgenic strains carrying low-copy extrachromosomal arrays. Extrachromosomal arrays were integrated by γ-irradiation with 50 Gy and F2 progeny were selected for 100% inheritance of the Rol-6 phenotype. Mapping analysis showed that the array was integrated in chromosome III.

**SSA reporter assay**

Worms were bleached to obtain synchronized L1 larvae. mCherry::I-SceI was induced by heatshock at 34˚C for 30 minutes or 60 minutes. Correct induction of mCherry::I-SceI was determined by visualizing mCherry epifluorescence. Two days after heatshock, L4 larvae/young adult worms were rinsed off plates, washed twice with water and dried in a speedyvac, before being fixed in acetone. LacZ staining was performed with X-gal solution (0.04% X-gal, 5 mM ferricyanide, 5 mM ferrocyanide, 0.3% formamide, 166 mM Na_2_HPO_4_, 33 mM NaH_2_PO_4_, 0.2 mM MgCl_2_, 0.004% SDS, 75 µg/ml kanamycin)

**qRT-PCR primers**

Sequence of qRT-PCR primers are as follows: *lin-61* tgctgacatgtgtgaaaatcagtt and catgggagtccacatcatacagtt; *egl-1* actcgggattttttgatgactctg and aaaaagtccagaagacgatggaag; tbg-1 attcaatccgctatctctcctgtt and tcattcgaagtggtttaagcatgt. All data was normalized to tubulin beta (*tbg-1*) expression levels.

**L1 larvae IR assay**

Assay was performed as in Bailly et al 2010 but L1 worms were obtained by bleaching rather than from growing populations using Millipore filters.

**References**

Ceol, C.J., Stegmeier, F., Harrison, M.M., and Horvitz, H.R. (2006). Identification and classification of genes that act antagonistically to let-60 Ras signaling in Caenorhabditis elegans vulval development. Genetics *173*, 709–726.

Cuppen, E., Gort, E., Hazendonk, E., Mudde, J., van de Belt, J., Nijman, I.J., Guryev, V., and Plasterk, R.H.A. (2007). Efficient target-selected mutagenesis in Caenorhabditis elegans: toward a knockout for every gene. Genome Res *17*, 649–658.
